# Supplementary material for: A marine photosynthetic microbial cell factory as a platform for spider silk production
Source: Commun Biol. 2020 Jul 8;3:357. doi: 10.1038/s42003-020-1099-6 (PMC7343832; doi:10.1038/s42003-020-1099-6)
Supplement: Supplementary file 1 — Supplementary Information [file 42003_2020_1099_MOESM1_ESM.pdf]

# A Marine Photosynthetic Microbial Cell Factory as A Platform for Spider Silk Production

Choon Pin Foong[1,2], Mieko Higuchi-Takeuchi[1], Ali D. Malay[1], Nur Alia Oktaviani[1], Chonprakun Thagun[1] and Keiji Numata[1,2]*

[1]Biomacromolecules Research Team, RIKEN Center for Sustainable Resource Science, 2-1 Hirosawa, Wako, Saitama 351-0198, Japan.

[2]Department of Material Chemistry, Graduate School of Engineering, Kyoto University, Kyoto-Daigaku-Katsura, Nishikyo-ku, Kyoto 615-8510, Japan.

Corresponding author: Keiji Numata, Email: keiji.numata@riken.jp

## Supplementary Figures

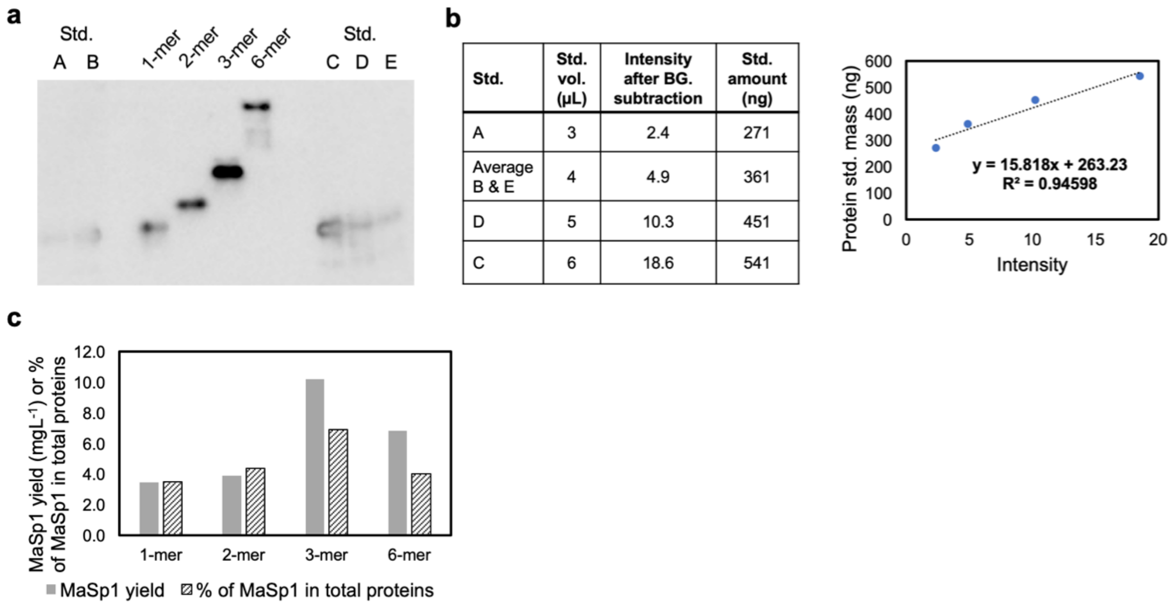


**Supplementary Fig. 1:** Semiquantitative measurement of MaSp1-(1-mer, 2-mer, 3-mer and 6-mer) expression in recombinant *R. sulfidophilum* under photoheterotrophic conditions (as in Fig. 1d). Recombinant *R. sulfidophilum* cells harboring pBBR1-$P_{trc}$-MaSp1-(1-mer, 2-mer, 3-mer and 6-mer) were cultivated in 50 mL centrifuge tubes containing 50 mL of marine broth with 100 mg $L^{-1}$ kanamycin at 30°C with continuous far-red LED light (730 nm, 20 to 30 W $m^{-2}$) for 4 days. **a,** Western blot using a monoclonal anti-His•Tag antibody, which targeted histidine-tagged MaSp1-(1-mer, 2-mer, 3-mer or 6-mer) proteins. **b**, Purified MaSp1-(1-mer) with a known concentration was used as a standard protein to generate a calibration curve. **c**, MaSp1 protein yield and percentage of MaSp1 in total proteins were quantified using Fiji/ImageJ version 1.52p and calculated based on a calibration curve. (Abbreviation: std. = standard; vol.= volume; BG. = background).

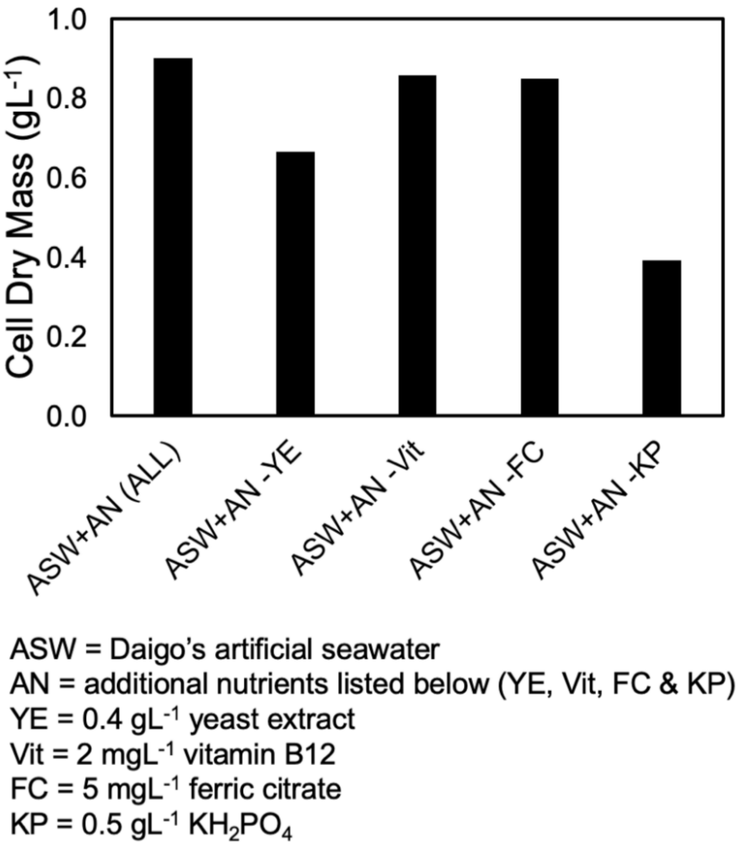


**Supplementary Fig. 2:** Evaluation of nutrients that could affect the growth of recombinant *R. sulfidophilum* in artificial seawater under photoautotrophic conditions. Recombinant *R. sulfidophilum* harboring pBBR1-$P_{trc}$-MaSp1-(1-mer) was cultivated using 20 mL of Daigo's artificial seawater (ASW) SP for marine microalgae medium in a 20 mL glass vial with a rubber stopper cap at 30°C with continuous far-red LED light (730 nm, 20 to 30 W $m^{-2}$) for 7 days. Inorganic carbon was supplied as 1 g $L^{-1}$ sodium bicarbonate, while nitrogen was supplied via daily nitrogen gas bubbling at 0.5 L $d^{-1}$. ASW contained 100 mg $L^{-1}$ kanamycin.

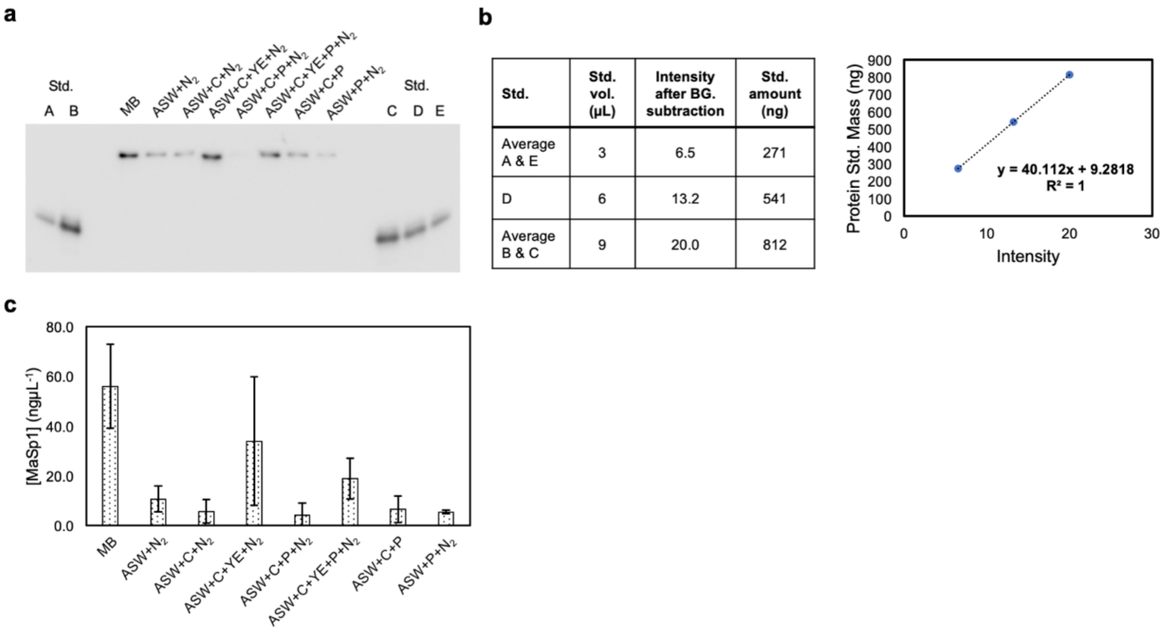


| Std. | Std. vol. (µL) | Intensity after BG. subtraction | Std. amount (ng) |
|---|---|---|---|
| Average A & E | 3 | 6.5 | 271 |
| D | 6 | 13.2 | 541 |
| Average B & C | 9 | 20.0 | 812 |

**Supplementary Fig. 3:** Semiquantitative measurement of MaSp1-(6-mer) expression in recombinant *R. sulfidophilum* under photoheterotrophic and photoautotrophic growth conditions (as in Fig. 2c-d). Recombinant *R. sulfidophilum* harboring pBBR1-$P_{trc}$-MaSp1-(6-mer) was cultivated using 20 mL of Daigo's artificial seawater (ASW) SP for marine microalgae medium in a 20 mL glass vial with a rubber stopper cap at 30°C with continuous far-red LED light (730 nm, 20 to 30 W $m^{-2}$) for 7 days. Inorganic carbon was supplied as 1 g $L^{-1}$ sodium bicarbonate, while nitrogen was supplied via daily nitrogen gas bubbling at 0.5 L $d^{-1}$. Marine broth (MB) and ASW contained 100 mg $L^{-1}$ kanamycin. **a**, Western blot using a monoclonal anti-His•Tag antibody, which targeted the histidine-tagged MaSp1 protein. **b**, Purified MaSp1-(1-mer) with a known concentration was used as a standard protein to generate a calibration curve plot. **c**, MaSp1-(6-mer) concentrations (mean values ± SD, n = 3 independent biological replicates) were quantified using Fiji/ImageJ version 1.52p and calculated based on a calibration curve. (Abbreviation: std. = standard; vol.= volume; BG. = background; C = $NaHCO_3$, YE = 0.4 g $L^{-1}$ yeast extract, $N_2$ = nitrogen gas and P = 0.5 g $L^{-1}$ $KH_2PO_4$).

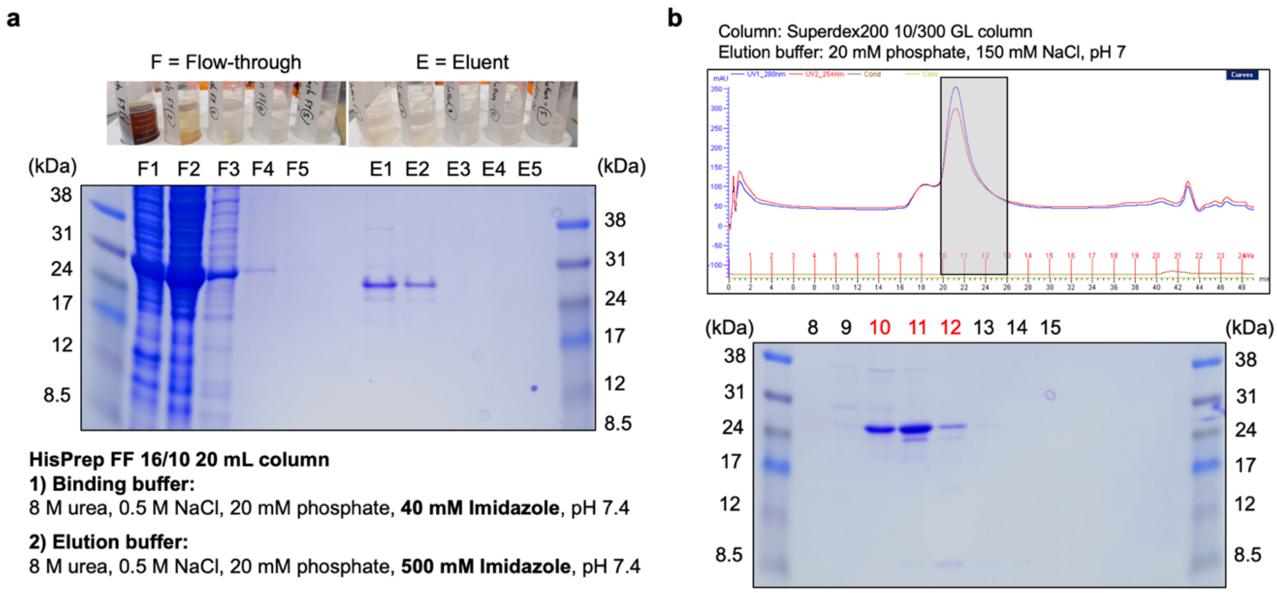


**Supplementary Fig. 4:** Purification of MaSp1-(6-mer) spidroin. **a**, His-Tag affinity chromatography. Soluble proteins were injected into a HisPrep™ FF 16/10 20 mL column, washed with binding buffer and eluted with elution buffer. E1 and E2 were combined and further concentrated using an Amicon Ultra-15 6 MWCO 3000 Da centrifugal filter. **b**, Gel-filtration chromatography. The concentrated eluent was further purified with an ÄKTAexplorer equipped with a Superdex®200 10/300 GL column. Fractions from tube numbers 10 to 12, which contained the appropriate size of MaSp1-(6-mer) protein, were combined and concentrated using an Amicon Ultra-15 6 MWCO 3000 Da centrifugal filter before a desalting step.

## Supplementary Tables

**Supplementary Table 1:** List of primers used for construction of the pBBR1-$P_{trc}$-MaSp1 plasmid

| **Primer** | **Nucleotide sequence (5' to 3')** |
|---|---|
| Trc-Prom_Fwd-*Xba*I | ATA TCTAGA TGC TTC TGG CGT CAG GCA G |
| Trc-Prom_Rev-RBS-*Eco*RI | AGC GAATTC **TCT CCT** ATT GTC TCT CTG CAC GTG C |
| MaSp1_Fwd-*Eco*RI | AGC GAATTC ATG CAC CAT CATCATCATCAT TCT |
| MaSp1_Rev-*Sal*I | AGT GTCGAC TTA ACT AGT CCC CTG AGA ACC CAG |
| MaSp1-Rep_Rev-*Sal*I | AGT GTCGAC TGG TGG TGG TGG TGC TGC |

Restriction enzyme digestion sites are underlined.

Bold letters represent the ribosome binding site (RBS) sequence **'AGGAGA'**

**Supplementary Table 2:** List of components and amino acid sequences in the MaSp1 protein constructs

| **Protein component** | **Amino acid sequence** |
|---|---|
| N-terminus | MHHHHHHSSG**LVPRG**SGM**KETAAAKFERQHMDS**PDLGT**DDDK**A |
| MaSp1-(1-mer) | MAA*SGRGGLGGQGAGAAAAAGGAGQGGYGGLGSQGT*S |
| MaSp1-(2-mer) | MAA*SGRGGLGGQGAGAAAAAGGAGQGGYGGLGSQGT* *SGRGGLGGQGAGAAAAAGGAGQGGYGGLGSQGT*S |
| MaSp1-(3-mer) | MAA*SGRGGLGGQGAGAAAAAGGAGQGGYGGLGSQGT* *SGRGGLGGQGAGAAAAAGGAGQGGYGGLGSQGT* *SGRGGLGGQGAGAAAAAGGAGQGGYGGLGSQGT*S |
| MaSp1-(6-mer) | MAA*SGRGGLGGQGAGAAAAAGGAGQGGYGGLGSQGT* *SGRGGLGGQGAGAAAAAGGAGQGGYGGLGSQGT* *SGRGGLGGQGAGAAAAAGGAGQGGYGGLGSQGT* *SGRGGLGGQGAGAAAAAGGAGQGGYGGLGSQGT* *SGRGGLGGQGAGAAAAAGGAGQGGYGGLGSQGT* *SGRGGLGGQGAGAAAAAGGAGQGGYGGLGSQGT*S |

6X His-Tag 'HHHHHH', thrombin cleavage site '**LVPRG**', S-Tag '**KETAAAKFERQHMDS**' and enterokinase cleavage site '**DDDK**'.

Italic letters represent the repetitive amino acid residues of MaSp1
