# Supplementary material for: A marine photosynthetic microbial cell factory as a platform for spider silk production
Source: Commun Biol. 2020 Jul 8;3:357. doi: 10.1038/s42003-020-1099-6 (PMC7343832; doi:10.1038/s42003-020-1099-6)
Supplement: Supplementary file 2 — Description of Additional Supplementary Files [file 42003_2020_1099_MOESM2_ESM.pdf]

**Description of Additional Supplementary Files**

File Name: Supplementary Data 1
Description: LC-MS/MS analysis of the excised bands of target proteins MaSp1-(1-mer, 2-mer, 3-mer and 6-mer) from SDS-PAGE gels (Fig. 1c). The protein bands were digested with trypsin, and the LC-MS/MS data were searched against in-house protein databases using the MASCOT program.
